# Bis(2,2′-bipyridine-κ^2^ *N*,*N*′)(croconato-κ^2^ *O*,*O*′)nickel(II)

**DOI:** 10.1107/S1600536808033771

**Published:** 2008-10-25

**Authors:** Hong-Feng Chen, Qi Fang, Wen-Tao Yu

**Affiliations:** a State Key Laboratory of Crystal Materials, Shandong University, Jinan 250100, Shandong Province, People’s Republic of China

## Abstract

The title compound, [Ni(C_5_O_5_)(C_10_H_8_N_2_)_2_], lies across a crystallographic twofold axis, around which two 2,2′-bipyridine (2,2′-bipy) ligands are arranged in a propeller manner. The local geometry of the NiN_4_O_2_ coordination core basically adopts an octa­hedral geometry. The mol­ecular twofold axis is along the direction of the mol­ecular dipole moment, and the complex is packed with its dipole moment alternately along the +*b* and −*b* directions. The crystal structure is stabilized by inter­molecular C—H⋯O hydrogen bonds.

## Related literature

For the synthesis, see: Chen *et al.* (2008 ▶). For related structures, see: Chen *et al.* (2005 ▶, 2007 ▶). For other related literature, see: Coronado *et al.* (2007 ▶); Wang *et al.* (2002 ▶).
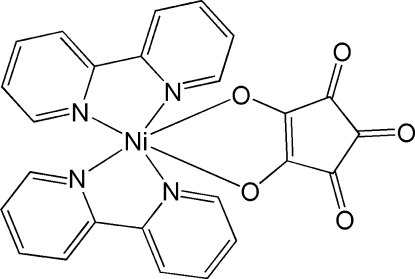


## Experimental

### 

#### Crystal data

[Ni(C_5_O_5_)(C_10_H_8_N_2_)_2_]
*M* *_r_* = 511.13
Orthorhombic, 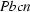

*a* = 12.725 (5) Å
*b* = 10.752 (5) Å
*c* = 15.733 (5) Å
*V* = 2152.6 (15) Å^3^
*Z* = 4
Mo *K*α radiation
μ = 0.95 mm^−1^
*T* = 293 (2) K
0.20 × 0.19 × 0.10 mm

#### Data collection

Bruker APEXII CCD diffractometer
Absorption correction: multi-scan (*APEX2*; Bruker, 2005 ▶) *T*_min_ = 0.821, *T*_max_ = 0.902
10055 measured reflections
2466 independent reflections
1536 reflections with *I* > 2σ(*I*)
*R*_int_ = 0.089

#### Refinement

*R*[*F* ^2^ > 2σ(*F* ^2^)] = 0.044
*wR*(*F* ^2^) = 0.102
*S* = 1.02
2466 reflections
160 parameters
H-atom parameters constrained
Δρ_max_ = 0.26 e Å^−3^
Δρ_min_ = −0.38 e Å^−3^

### 

Data collection: *APEX2* (Bruker, 2005 ▶); cell refinement: *APEX2*; data reduction: *APEX2*; program(s) used to solve structure: *SHELXS97* (Sheldrick, 2008 ▶); program(s) used to refine structure: *SHELXL97* (Sheldrick, 2008 ▶); molecular graphics: *SHELXTL* (Sheldrick, 2008 ▶); software used to prepare material for publication: *WinGX* (Farrugia, 1999 ▶).

## Supplementary Material

Crystal structure: contains datablocks global, I. DOI: 10.1107/S1600536808033771/lx2071sup1.cif

Structure factors: contains datablocks I. DOI: 10.1107/S1600536808033771/lx2071Isup2.hkl

Additional supplementary materials:  crystallographic information; 3D view; checkCIF report

## Figures and Tables

**Table 1:** Hydrogen-bond geometry (Å, °)

*D*—H⋯*A*	*D*—H	H⋯*A*	*D*⋯*A*	*D*—H⋯*A*
C4—H4⋯O1^i^	0.93	2.57	3.340 (4)	141
C8—H8⋯O2^ii^	0.93	2.24	3.114 (4)	156
C9—H9⋯O3^iii^	0.93	2.57	3.222 (4)	127

Symmetry codes: (i) 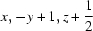; (ii) 
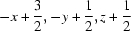
; (iii) 
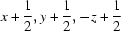
.

**Fig. 1.**
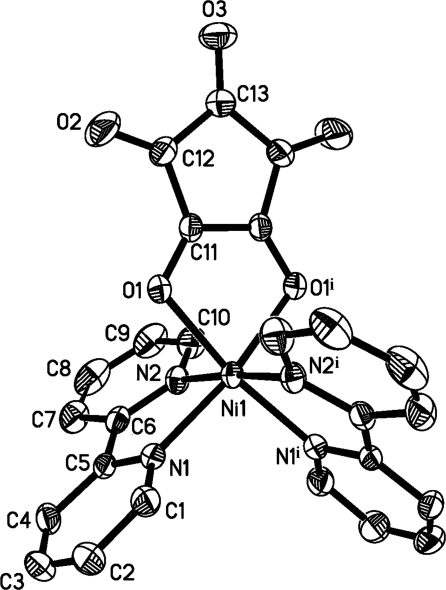
The molecular structure of [Ni(C5O5) (2,2'-bipy)2]. Displacement ellipsoids are drawn at the 30% probability level and H atoms have been omitted. [symmetry code: (i) -x + 1, y, -z + 3/2.]

## supplementary crystallographic information

### Comment

The croconate C_5_O_5_^2-^ anion has attracted increasing attention in recent years because this polydentate ligand gave rise to a variety of interesting complexes (Chen *et al.*, 2008; Coronado *et al.*, 2007; Chen *et al.*, 2005; Wang *et al.*, 2002;). Typically, the C_5_O_5_^2-^ anion serves as a terminal bidentate chelate ligand or a bridging ligand utilizing more than two O atoms for coordination. We previously reported a mixing-coordinated complex [Ni(C_5_O_5_)(phen)_2_] with 1,10-phenanthroline (phen) as the first ligand and the C_5_O_5_^2-^ anion as the second ligand (Chen *et al.*, 2007). The similar chelating behavior of 2,2'-bipy and 1,10-phen prompted us to replace phen ligand by 2,2'-bipy. In this report, the structure of this mixing-coordinated complex is reported.

The title compound crystalizes to the same space group as [Ni(C_5_O_5_)(phen)_2_]. Both crystals have very similar cell parameters and show many common features. The chiral molecule lies across twofold axis which is along the direction of the molecular dipole moment. Around the molecular axis, two 2,2'-bipy ligands are arranged in a propeller manner. The Ni^2+^ is coordinated by four N atoms of the two 2,2'-bipy ligands and two O atoms of a croconate ligand to furnish a slightly distored octahedral NiN_4_O_2_ coordination core. The dihedral angle between the croconate plane and a 2,2'-bipy plane is 88.7 (1)°, and that between the two 2,2'-bipy planes is 81.9 (1)° in [Ni(C_5_O_5_) (2,2'-bipy)_2_]. These are close to the corresponding dihedral angles (86.9 (1)° and 86.6 (1)°) in [Ni(C_5_O_5_)(phen)_2_]. The C—O bond lengths involving coordinated O atoms are longer than those of other C—O bonds. In both crystals, molecules packed alternately along +b and-b directions.

However, we can not fail to notice some differences between the two crystals. The Ni—O band length of [Ni(C_5_O_5_)(2,2'-bipy)_2_] {2.102 (2) Å} is longer than that {2.098 (3) Å} of [Ni(C_5_O_5_)(phen)_2_]. Meanwhile, the Ni—N band lengths of [Ni(C_5_O_5_)(2,2'-bipy)_2_] {2.059 (2), 2.066 (2) Å} is considerably shorter than those {2.071 (3), 2.088 (3) Å} of [Ni(C_5_O_5_)(phen)_2_]. It seems that 2,2'-bipyridine is a stronger ligand to Ni^2+^ in comparison with 1,10-phenanthroline. The crystal structure is stabilized by intermolecular C—H···O hydrogen bonds (Table 1).

### Experimental

[K_2_(C_5_O_5_)] (0.050 g, 0.23 mmol) and NiCl_2_.6H_2_O (0.060 g, 0.25 mmol) were dissolved in mixed solvent of water (15 ml) and dimethylformamide (10 ml). Then 2,2'-bipy (0.080 g, 0.51 mmol) was added. The mixture was heated to 340–350 K under continuous stirring for 20 min and then filtered. The green-yellow prisms crystals were obtained by slow evaporation at 313 K.

### Refinement

All H atoms were positioned geometrically and allowed to ride on their attached atom. The C—H bond lengths for aromatic groups were set to 0.93 Å.

### Crystal data

**Table:**

[Ni(C_5_O_5_)(C_10_H_8_N_2_)_2_]	*F*(000) = 1048
*M**_r_* = 511.13	*D*_x_ = 1.577 Mg m^−^^3^
Orthorhombic, *P**b**c**n*	Mo *K*α radiation, λ = 0.71069 Å
Hall symbol: -P_2n 2ab	Cell parameters from 2518 reflections
*a* = 12.725 (5) Å	θ = 2.5–23.4°
*b* = 10.752 (5) Å	µ = 0.95 mm^−^^1^
*c* = 15.733 (5) Å	*T* = 293 K
*V* = 2152.6 (15) Å^3^	Prism, green-yellow
*Z* = 4	0.20 × 0.19 × 0.10 mm

### Data collection

**Table:**

Bruker APEXII CCD diffractometer	2466 independent reflections
Radiation source: fine-focus sealed tube	1536 reflections with *I* > 2σ(*I*)
graphite	*R*_int_ = 0.089
Detector resolution: 10.0 pixels mm^-1^	θ_max_ = 27.5°, θ_min_ = 2.5°
φ and ω scans	*h* = −15→16
Absorption correction: multi-scan (APEX2; Bruker, 2005)	*k* = −13→10
*T*_min_ = 0.821, *T*_max_ = 0.902	*l* = −20→19
10055 measured reflections	

### Refinement

**Table:**

Refinement on *F*^2^	Primary atom site location: structure-invariant direct methods
Least-squares matrix: full	Secondary atom site location: difference Fourier map
*R*[*F*^2^ > 2σ(*F*^2^)] = 0.044	Hydrogen site location: inferred from neighbouring sites
*wR*(*F*^2^) = 0.102	H-atom parameters constrained
*S* = 1.02	*w* = 1/[σ^2^(*F*_o_^2^) + (0.0372*P*)^2^ + 0.0108*P*] where *P* = (*F*_o_^2^ + 2*F*_c_^2^)/3
2466 reflections	(Δ/σ)_max_ < 0.001
160 parameters	Δρ_max_ = 0.26 e Å^−^^3^
0 restraints	Δρ_min_ = −0.38 e Å^−^^3^

### Special details

**Table:**

Geometry. All esds (except the esd in the dihedral angle between two l.s. planes) are estimated using the full covariance matrix. The cell esds are taken into account individually in the estimation of esds in distances, angles and torsion angles; correlations between esds in cell parameters are only used when they are defined by crystal symmetry. An approximate (isotropic) treatment of cell esds is used for estimating esds involving l.s. planes.
Refinement. Refinement of F^2^ against ALL reflections. The weighted R-factor wR and goodness of fit S are based on F^2^, conventional R-factors R are based on F, with F set to zero for negative F^2^. The threshold expression of F^2^ > 2sigma(F^2^) is used only for calculating R-factors(gt) etc. and is not relevant to the choice of reflections for refinement. R-factors based on F^2^ are statistically about twice as large as those based on F, and R- factors based on ALL data will be even larger.

### Fractional atomic coordinates and isotropic or equivalent isotropic displacement parameters (Å^2^)

**Table:**

	*x*	*y*	*z*	*U*_iso_*/*U*_eq_	
Ni1	0.5000	0.46011 (4)	0.2500	0.03546 (17)	
O1	0.56048 (15)	0.31353 (15)	0.17633 (10)	0.0439 (5)	
O2	0.6016 (2)	0.0512 (2)	0.12408 (16)	0.1053 (10)	
O3	0.5000	−0.1097 (3)	0.2500	0.0700 (9)	
N1	0.45364 (18)	0.58822 (19)	0.34037 (12)	0.0397 (5)	
N2	0.63184 (17)	0.46676 (19)	0.32573 (12)	0.0419 (6)	
C1	0.3650 (3)	0.6535 (3)	0.34092 (17)	0.0531 (8)	
H1	0.3173	0.6410	0.2970	0.064*	
C2	0.3402 (3)	0.7384 (3)	0.4029 (2)	0.0717 (10)	
H2	0.2774	0.7824	0.4013	0.086*	
C3	0.4114 (4)	0.7561 (3)	0.4673 (2)	0.0742 (11)	
H3	0.3977	0.8142	0.5097	0.089*	
C4	0.5025 (3)	0.6888 (3)	0.46926 (18)	0.0570 (9)	
H4	0.5502	0.6989	0.5135	0.068*	
C5	0.5226 (2)	0.6052 (2)	0.40425 (15)	0.0406 (7)	
C6	0.6200 (2)	0.5320 (2)	0.39824 (16)	0.0431 (7)	
C7	0.6964 (3)	0.5264 (3)	0.4613 (2)	0.0638 (9)	
H7	0.6872	0.5696	0.5119	0.077*	
C8	0.7854 (3)	0.4569 (4)	0.4486 (2)	0.0770 (12)	
H8	0.8367	0.4526	0.4906	0.092*	
C9	0.7982 (2)	0.3940 (3)	0.3737 (2)	0.0679 (10)	
H9	0.8587	0.3479	0.3633	0.081*	
C10	0.7195 (2)	0.4008 (3)	0.31437 (19)	0.0575 (8)	
H10	0.7276	0.3572	0.2637	0.069*	
C11	0.5300 (2)	0.2129 (3)	0.21214 (15)	0.0393 (6)	
C12	0.5511 (3)	0.0864 (3)	0.18605 (18)	0.0530 (8)	
C13	0.5000	0.0035 (4)	0.2500	0.0487 (10)	

### Atomic displacement parameters (Å^2^)

**Table:**

	*U*^11^	*U*^22^	*U*^33^	*U*^12^	*U*^13^	*U*^23^
Ni1	0.0404 (3)	0.0388 (3)	0.0271 (2)	0.000	−0.0053 (2)	0.000
O1	0.0587 (13)	0.0418 (11)	0.0310 (9)	−0.0040 (10)	0.0083 (9)	0.0013 (8)
O2	0.167 (3)	0.0586 (15)	0.0903 (17)	−0.0026 (15)	0.0769 (19)	−0.0177 (13)
O3	0.086 (2)	0.0405 (17)	0.084 (2)	0.000	0.0118 (18)	0.000
N1	0.0510 (14)	0.0360 (12)	0.0322 (11)	−0.0008 (12)	−0.0020 (11)	0.0015 (10)
N2	0.0437 (14)	0.0480 (14)	0.0340 (12)	0.0003 (12)	−0.0082 (10)	0.0017 (10)
C1	0.064 (2)	0.0507 (18)	0.0443 (16)	0.0138 (17)	0.0003 (16)	0.0011 (14)
C2	0.098 (3)	0.057 (2)	0.060 (2)	0.024 (2)	0.022 (2)	−0.0018 (17)
C3	0.119 (3)	0.048 (2)	0.056 (2)	−0.002 (2)	0.028 (2)	−0.0121 (16)
C4	0.084 (2)	0.0514 (17)	0.0359 (15)	−0.022 (2)	0.0095 (16)	−0.0076 (13)
C5	0.0582 (19)	0.0341 (14)	0.0295 (13)	−0.0130 (14)	0.0012 (12)	0.0026 (11)
C6	0.0535 (18)	0.0442 (16)	0.0317 (14)	−0.0198 (15)	−0.0080 (13)	0.0070 (13)
C7	0.076 (2)	0.068 (2)	0.0482 (18)	−0.023 (2)	−0.0272 (17)	0.0038 (15)
C8	0.065 (2)	0.090 (3)	0.076 (3)	−0.023 (2)	−0.041 (2)	0.033 (2)
C9	0.0442 (18)	0.080 (3)	0.079 (2)	0.0036 (19)	−0.0144 (18)	0.026 (2)
C10	0.0494 (19)	0.067 (2)	0.0555 (19)	0.0078 (18)	−0.0081 (15)	0.0055 (16)
C11	0.0431 (17)	0.0439 (16)	0.0309 (13)	−0.0025 (14)	−0.0005 (11)	−0.0019 (12)
C12	0.066 (2)	0.0462 (17)	0.0463 (17)	−0.0038 (17)	0.0129 (16)	−0.0085 (14)
C13	0.052 (2)	0.042 (2)	0.052 (2)	0.000	0.000 (2)	0.000

### Geometric parameters (Å, °)

**Table:**

Ni1—N2	2.059 (2)	C3—C4	1.367 (4)
Ni1—N2^i^	2.059 (2)	C3—H3	0.9300
Ni1—N1	2.066 (2)	C4—C5	1.385 (4)
Ni1—N1^i^	2.066 (2)	C4—H4	0.9300
Ni1—O1^i^	2.1022 (18)	C5—C6	1.472 (4)
Ni1—O1	2.1022 (18)	C6—C7	1.389 (4)
O1—C11	1.280 (3)	C7—C8	1.371 (5)
O2—C12	1.227 (3)	C7—H7	0.9300
O3—C13	1.217 (5)	C8—C9	1.369 (5)
N1—C1	1.328 (3)	C8—H8	0.9300
N1—C5	1.347 (3)	C9—C10	1.370 (4)
N2—C10	1.334 (3)	C9—H9	0.9300
N2—C6	1.347 (3)	C10—H10	0.9300
C1—C2	1.372 (4)	C11—C11^i^	1.415 (5)
C1—H1	0.9300	C11—C12	1.446 (4)
C2—C3	1.373 (5)	C12—C13	1.493 (4)
C2—H2	0.9300	C13—C12^i^	1.493 (4)
			
N2—Ni1—N2^i^	176.02 (12)	C3—C4—C5	118.9 (3)
N2—Ni1—N1	79.12 (9)	C3—C4—H4	120.6
N2^i^—Ni1—N1	98.19 (8)	C5—C4—H4	120.6
N2—Ni1—N1^i^	98.19 (8)	N1—C5—C4	121.3 (3)
N2^i^—Ni1—N1^i^	79.12 (9)	N1—C5—C6	115.4 (2)
N1—Ni1—N1^i^	96.35 (11)	C4—C5—C6	123.3 (3)
N2—Ni1—O1^i^	90.30 (8)	N2—C6—C7	120.2 (3)
N2^i^—Ni1—O1^i^	92.68 (8)	N2—C6—C5	115.3 (2)
N1—Ni1—O1^i^	90.91 (8)	C7—C6—C5	124.5 (3)
N1^i^—Ni1—O1^i^	169.72 (7)	C8—C7—C6	119.8 (3)
N2—Ni1—O1	92.68 (8)	C8—C7—H7	120.1
N2^i^—Ni1—O1	90.30 (8)	C6—C7—H7	120.1
N1—Ni1—O1	169.72 (7)	C9—C8—C7	119.5 (3)
N1^i^—Ni1—O1	90.91 (8)	C9—C8—H8	120.2
O1^i^—Ni1—O1	82.88 (10)	C7—C8—H8	120.2
C11—O1—Ni1	106.29 (15)	C8—C9—C10	118.2 (3)
C1—N1—C5	118.5 (2)	C8—C9—H9	120.9
C1—N1—Ni1	126.82 (19)	C10—C9—H9	120.9
C5—N1—Ni1	114.72 (18)	N2—C10—C9	123.2 (3)
C10—N2—C6	118.9 (2)	N2—C10—H10	118.4
C10—N2—Ni1	125.83 (19)	C9—C10—H10	118.4
C6—N2—Ni1	114.66 (19)	O1—C11—C11^i^	122.26 (14)
N1—C1—C2	123.5 (3)	O1—C11—C12	127.9 (2)
N1—C1—H1	118.3	C11^i^—C11—C12	109.83 (15)
C2—C1—H1	118.3	O2—C12—C11	127.8 (3)
C1—C2—C3	117.7 (3)	O2—C12—C13	125.4 (3)
C1—C2—H2	121.1	C11—C12—C13	106.8 (2)
C3—C2—H2	121.1	O3—C13—C12^i^	126.64 (17)
C4—C3—C2	120.2 (3)	O3—C13—C12	126.64 (17)
C4—C3—H3	119.9	C12^i^—C13—C12	106.7 (3)
C2—C3—H3	119.9		
			
N2—Ni1—O1—C11	90.49 (17)	C1—N1—C5—C6	−178.6 (2)
N2^i^—Ni1—O1—C11	−92.14 (18)	Ni1—N1—C5—C6	1.2 (3)
N1—Ni1—O1—C11	53.7 (5)	C3—C4—C5—N1	−1.0 (4)
N1^i^—Ni1—O1—C11	−171.27 (17)	C3—C4—C5—C6	177.3 (3)
O1^i^—Ni1—O1—C11	0.52 (13)	C10—N2—C6—C7	−2.5 (4)
N2—Ni1—N1—C1	174.7 (2)	Ni1—N2—C6—C7	169.1 (2)
N2^i^—Ni1—N1—C1	−2.3 (2)	C10—N2—C6—C5	178.1 (2)
N1^i^—Ni1—N1—C1	77.5 (2)	Ni1—N2—C6—C5	−10.3 (3)
O1^i^—Ni1—N1—C1	−95.2 (2)	N1—C5—C6—N2	6.1 (3)
O1—Ni1—N1—C1	−147.8 (4)	C4—C5—C6—N2	−172.3 (2)
N2—Ni1—N1—C5	−5.04 (17)	N1—C5—C6—C7	−173.3 (2)
N2^i^—Ni1—N1—C5	177.93 (18)	C4—C5—C6—C7	8.3 (4)
N1^i^—Ni1—N1—C5	−102.20 (19)	N2—C6—C7—C8	1.8 (4)
O1^i^—Ni1—N1—C5	85.09 (18)	C5—C6—C7—C8	−178.8 (3)
O1—Ni1—N1—C5	32.5 (5)	C6—C7—C8—C9	0.2 (5)
N1—Ni1—N2—C10	179.3 (2)	C7—C8—C9—C10	−1.5 (5)
N1^i^—Ni1—N2—C10	−85.7 (2)	C6—N2—C10—C9	1.2 (4)
O1^i^—Ni1—N2—C10	88.5 (2)	Ni1—N2—C10—C9	−169.4 (2)
O1—Ni1—N2—C10	5.6 (2)	C8—C9—C10—N2	0.8 (5)
N1—Ni1—N2—C6	8.43 (17)	Ni1—O1—C11—C11^i^	−1.5 (4)
N1^i^—Ni1—N2—C6	103.37 (17)	Ni1—O1—C11—C12	−179.9 (2)
O1^i^—Ni1—N2—C6	−82.44 (17)	O1—C11—C12—O2	−0.7 (5)
O1—Ni1—N2—C6	−165.32 (17)	C11^i^—C11—C12—O2	−179.3 (3)
C5—N1—C1—C2	0.6 (4)	O1—C11—C12—C13	178.9 (2)
Ni1—N1—C1—C2	−179.1 (2)	C11^i^—C11—C12—C13	0.3 (4)
N1—C1—C2—C3	0.1 (5)	O2—C12—C13—O3	−0.6 (4)
C1—C2—C3—C4	−1.2 (5)	C11—C12—C13—O3	179.89 (13)
C2—C3—C4—C5	1.7 (5)	O2—C12—C13—C12^i^	179.4 (4)
C1—N1—C5—C4	−0.1 (4)	C11—C12—C13—C12^i^	−0.11 (13)
Ni1—N1—C5—C4	179.63 (19)		

Symmetry codes: (i) −*x*+1, *y*, −*z*+1/2.

### Hydrogen-bond geometry (Å, °)

**Table:**

*D*—H···*A*	*D*—H	H···*A*	*D*···*A*	*D*—H···*A*
C4—H4···O1^ii^	0.93	2.57	3.340 (4)	141
C8—H8···O2^iii^	0.93	2.24	3.114 (4)	156
C9—H9···O3^iv^	0.93	2.57	3.222 (4)	127

Symmetry codes: (ii) *x*, −*y*+1, *z*+1/2; (iii) −*x*+3/2, −*y*+1/2, *z*+1/2; (iv) *x*+1/2, *y*+1/2, −*z*+1/2.
